# Depth-Resolved Visualization of Perifoveal Retinal Vasculature in Preterm Infants Using Handheld Optical Coherence Tomography Angiography

**DOI:** 10.1167/tvst.10.9.10

**Published:** 2021-08-06

**Authors:** Pujan R. Patel, Ryan Imperio, Christian Viehland, Du Tran-Viet, Stephanie J. Chiu, Vincent Tai, Joseph A. Izatt, Cynthia A. Toth, Xi Chen, for the BabySTEPS Group

**Affiliations:** 1 Department of Ophthalmology, Duke University, Durham, NC, USA; 2 Drexel University College of Medicine, Philadelphia, PA, USA; 3 Department of Biomedical Engineering, Duke University, Durham, NC, USA

**Keywords:** optical coherence tomography angiography (OCT-A), retinal vascular development, depth-resolved imaging, preterm infant, three-layered perifoveal vasculature

## Abstract

**Purpose:**

To establish methods to visualize depth-resolved perifoveal retinal vasculature in preterm infants using handheld optical coherence tomography angiography (OCT-A).

**Methods:**

In this exploratory study, eyes of preterm infants were imaged using an investigational noncontact, handheld swept-source OCT-A device as part of the prospective BabySTEPS infant retinal imaging study. We selected high-quality OCT-A volumes at two developmental stages for analysis. Customized MATLAB scripts were used to segment retinal layers, test offset parameters, and generate depth-resolved OCT-A slabs. The superficial (SCP), intermediate (ICP), and deep (DCP) capillary plexuses were visualized and qualitatively assessed by three image graders.

**Results:**

Six eyes from six preterm infants were included in this analysis. A three-layered perifoveal retinal vasculature was successfully visualized in all three eyes (three infants) in the 40 weeks postmenstrual age (PMA) group (one of three eyes with treated type 1 retinopathy of prematurity [ROP]). No obvious ICP or DCP was found in good-quality scans of the three eyes (three infants) in the 35 weeks PMA group (three of three eyes developed type 1 ROP).

**Conclusions:**

Custom segmentation parameters are useful to visualize perifoveal retinal vasculature in preterm infants. At term age, a three-layered capillary structure is visible in most eyes, while prior to detectable flow within the ICP and DCP, the perifoveal vasculature may be better visualized in two layers.

**Translational Relevance:**

Development of segmentation parameters for depth-resolved OCT-A of perifoveal retinal vasculature in preterm infants facilitates the study of human retinal vascular development and vascular pathologies of ROP.

## Introduction

The human retinal vasculature undergoes dynamic development in the second and third trimesters in utero. Formation of the primary retinal vasculature begins at the optic disc around 14 to 15 weeks’ gestation and expands outward in a four-lobe configuration to supply the avascular retina.^1^^,^^2^ In addition to peripheral expansion, the primary vasculature sprouts deeper into the retinal layers at around 26 to 28 weeks’ gestation and forms the secondary retinal vasculature.^3^ In primates, compared to the retinal vasculature stemming from the optic disc, the perifoveal vasculature forms slower and later in time and may not complete until 1 month after term birth.^4^^,^^5^ The retinal vasculature develops into three distinct layers: the superficial capillary plexus (SCP, from the primary retinal vasculature), the intermediate capillary plexus (ICP), and the deep capillary plexus (DCP), the latter two of which are derived from the secondary retinal vasculature.^6^^–^^8^ Our current understanding of the infant retinal and perifoveal vasculature relies on histopathologic studies of rare postmortem fetal eyes^1^^,^^3^^,^^9^ and by inference from histologic studies of nonhuman primates^3^^,^^4^^,^^10^ and other animals that possess an area centralis.^11^^,^^12^ Although retinal vascular development is well characterized in nonhuman primates, observation of such a process in humans is extremely limited, especially during late gestational stages.

The advent of handheld optical coherence tomography angiography (OCT-A) and its adaptation to bedside use has provided us an opportunity to noninvasively observe retinal vascular flow in the eyes of preterm infants.^13^^–^^17^ As much of the retinal vascular development of preterm infants occurs following parturition, handheld OCT-A holds promise to directly image the development of perifoveal retinal vasculature. Indeed, we have thus far imaged the formation of perifoveal vasculature in an infant who developed retinopathy of prematurity (ROP) and an infant with aggressive posterior ROP treated with bevacizumab.^13^^,^^18^^,^^19^ However, handheld OCT-A remains technically challenging. Other than limited reports,^15^^–^^17^^,^^20^ current uses of investigational handheld OCT-A in preterm infant eyes have been performed without depth resolution. The development of an infant-specific OCT-A segmentation algorithm is therefore a first step toward understanding human retinal vascular development through depth-resolved imaging.

In this study, we used an investigational handheld OCT-A device to obtain high-quality OCT-A images of the perifoveal retinal vasculature in six preterm infants at two distinct developmental time points. We used semiautomated retinal layer segmentation with manual correction and customized software to achieve depth-resolved visualization of the developing perifoveal retinal vasculature. Our study suggests that it is possible to visualize the three-layered perifoveal retinal capillaries in preterm infants, and varying offset parameters are needed to accommodate different stages of retinal vascular development.

## Methods

This is a secondary analysis of selected OCT-A images obtained from the prospective observational BabySTEPS infant retinal imaging study (NCT02887157). The study was approved by the Duke University Health System Institutional Review Board and adhered to the Health Insurance Portability and Accountability Act and the tenets of the Declaration of Helsinki.

### Participants

Premature infants were eligible for enrollment if they did not have severe neurologic or ocular comorbidities and underwent ROP screening at Duke University Hospital or required a clinically indicated examination under anesthesia (EUA) with written informed consent from parent or legal guardian. OCT-A images of the infants were obtained either nonsedated at the bedside or during EUA.

### OCT-A Imaging

OCT-A images were acquired using the investigational swept-source laser at 200 kHz and an OCT probe customized for handheld imaging (Duke University, Durham, NC, Supplementary Figure S1) at the bedside or during EUA with pharmacologic pupillary dilation.^13^ Each OCT-A volume was captured at 500 A-scans per B-scan and 250 or 500 B-scans per volume repeated four times at each lateral location (scan size equivalent to 2.5 × 5 mm or 5 × 5 mm in adult eyes). The image acquisition time was approximately 3.1 seconds or 6.2 seconds per volume.

### OCT-A Image Selection and Analysis

OCT-A imaging volumes were processed using speckle variance^21^ and segmented with custom software (Duke OCT Retinal Analysis Program [DOCTRAP] 65.6) (MATLAB R2017b; MathWorks, Natick, MA, USA) optimized for infant retinal image processing, and preliminary OCT-A previews were generated using MATLAB scripts. Approximately 38% of OCT-A images obtained during EUA and 91% of all OCT-A images obtained at the bedside were considered poor quality and inadequate for further analysis. Six eyes from six participants with high-quality image volumes were selected by experienced infant OCT-A graders (RI and XC) and included in this analysis. The image selection was based on volumes that include the fovea with clear retinal vascular patterns and minimal motion artifacts. Three imaging volumes were selected at around term age (40 weeks postmenstrual age [PMA]), while the other three were selected at an earlier time point (35 weeks PMA) with the goal to depict an earlier phase of retinal vascular formation.

The automatically segmented B-scan images were reviewed and manually corrected by experienced infant OCT-A graders (RI and XC). Customized MATLAB scripts were then used to test offset parameters using the retinal layer boundaries and to generate isolated en face slabs for the SCP, ICP, and DCP as described below. Because the pixel depth of the B-scan was 4.37 µm/pixel, offset parameters were tested in increments of one pixel (i.e., 4.37 µm).

1. SCP inner boundary was fixed at the vitreous/inner limiting membrane (ILM) junction.
2. SCP/ICP boundary ranged from 0 to 17.48 µm (0 to +4 pixels) above the inner plexiform layer (IPL)/inner nuclear layer (INL) junction.
3. ICP/DCP boundary was assessed via two reference layers:
  a. IPL/INL junction—offset ranged from 4.37 to 21.85 µm (−1 to −5 pixels) below the IPL/INL junction.
  b. Outer plexiform layer (OPL)/outer nuclear layer (ONL) junction—offset ranged from 21.85 to 43.7 µm (+5 to +10 pixels) above the OPL/ONL junction.
4. DCP outer boundary was fixed at the OPL/ONL junction.

For each offset parameter, the SCP, ICP, and DCP were visualized and qualitatively assessed. Determination of the optimal offset parameters for retinal capillary segmentation was performed by assessing the en face SCP/ICP and ICP/DCP slabs through sequential comparison, side-by-side/juxtaposition, and superimposition and by consensus of three OCT-A image graders (PRP, RI, XC). Inspection of ICP was given precedence over SCP because the SCP slab was more resilient than the ICP slab to the various offsets. The ICP and DCP slabs were optimized by minimizing the ICP projections onto the DCP. An example ICP collage with all tested offset combinations by juxtaposition is shown in Supplementary Figure S2.

All slabs were normalized so that the pixel values ranged from 0 to 1 and then thresholded to preserve intensities below 0.25. Contrast-limited adaptive histogram equalization (adapthisteq command in MATLAB) was then applied independently to each image row using a parameter of eight tiles across each row. To generate a three-colored OCT-A overlay, the “subtract” blend mode featured in Photoshop CC 2020 v21.2 (Adobe, Inc., San Jose, CA, USA) was used to remove SCP shadows from the ICP slab. Next, the SCP, ICP, and DCP slabs were colored yellow, cyan, and magenta, respectively, using a gradient map. The “lighten” blend mode was used to superimpose the three slabs for display.

## Results

Six OCT-A imaging volumes from six preterm infants acquired between 34 and 40 weeks PMA were included in this analysis. The participants were divided into two groups: three participants were at 35 ± 1 weeks and the other three were at 40 ± 1 weeks PMA. The participants’ gestational age at birth ranged from 23 to 33 weeks. Two out of six participants were male. One out of six eyes had macular edema. One out of the three eyes in the 40 weeks PMA group had treated type 1 ROP, while the other two did not have ROP. All three eyes in the 35 weeks PMA group developed type 1 ROP. The participant demographic and clinical characteristics are summarized in Table.

**Table.** Demographic and Clinical Characteristics of the Participants Included in the Study

Patient	Laterality	Gender	Gestational Age at Birth, wk	Postmenstrual Age at Imaging, wk	Fundus Appearance at Imaging	Macular Edema
1	OS	Female	24	40	Zone II stage 4A ROP following peripheral laser ablation	No
2	OS	Female	33	40	Vitreous hemorrhage, no ROP	No
3	OD	Female	26	39	Zone III stage 0 ROP	Yes
4	OS	Male	23	34	Zone II stage 3 ROP with plus disease	No
5	OS	Female	24	36	Zone I stage 2 ROP without plus disease	No
6	OS	Male	26	35	Zone II stage 3 ROP with plus disease	No

OD, right eye; OS, left eye; ROP, retinopathy of prematurity.

### Retinal Capillary Visualization at Term-Equivalent Age

**Figure 1.**
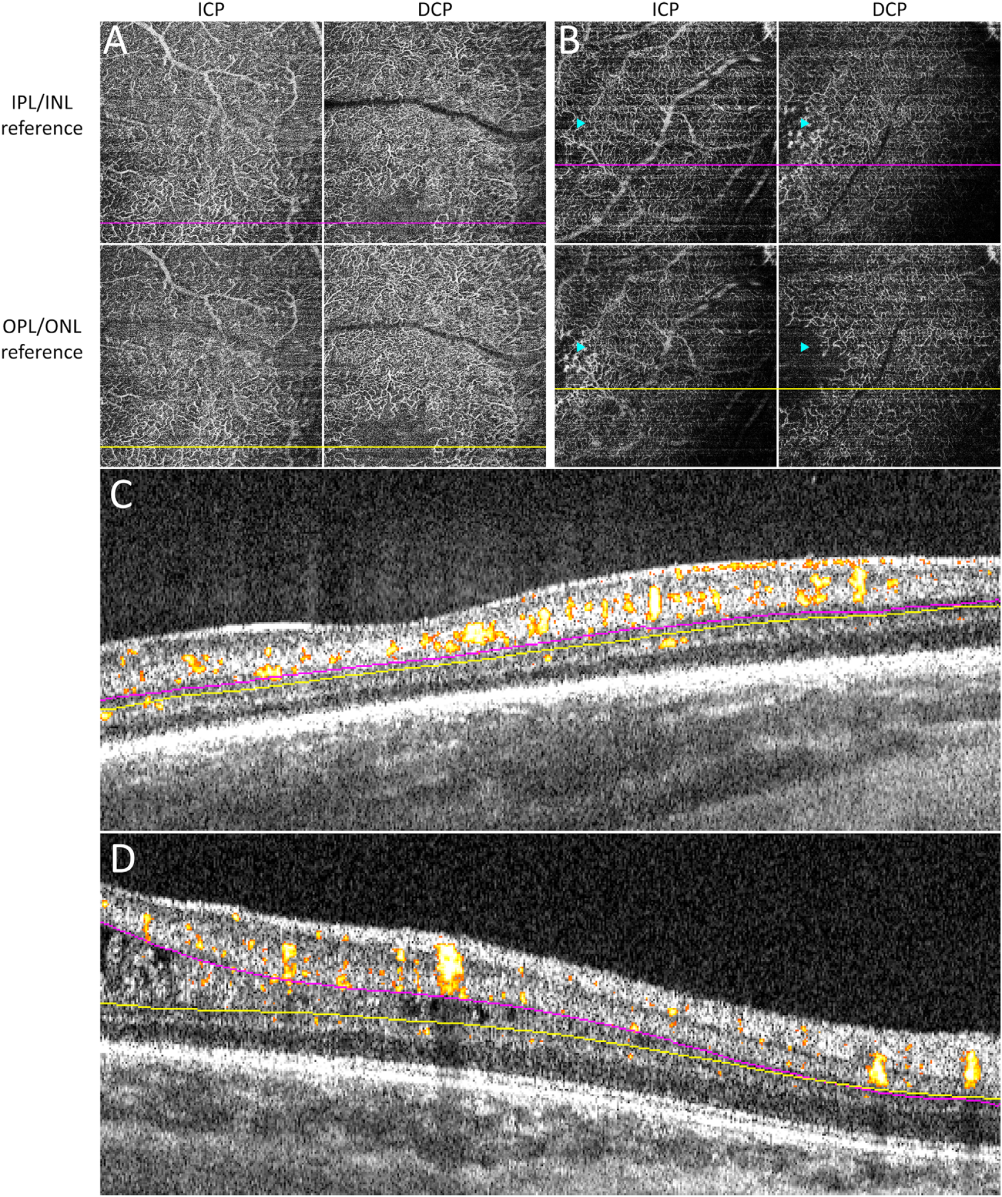
Comparison of using IPL/INL junction and OPL/ONL junction as the reference boundary for segmentation of the ICP and DCP. The reference boundary using the IPL/INL junction (ICP: from 8.74 µm above to 17.48 µm below the IPL/INL junction; DCP: 17.48 µm below the IPL/INL junction to the OPL/ONL junction) was compared to the reference boundary using the OPL/ONL junction (ICP: from 8.74 µm above the IPL/INL junction to 34.96 µm above the OPL/ONL junction; DCP: from 34.96 µm above to at the OPL/ONL junction). In an infant eye without macular edema, no obvious difference in ICP and DCP vascular patterns was observed between the two reference boundaries (A). In contrast, in the infant eye with macular edema, the ICP and DCP slabs showed different vascular patterns in the area of macular edema (B, *arrowheads*). In the cross-sectional B-scan with flow overlay, the optimal segmentation lines using the IPL/INL junction as reference (C, *magenta*) and the OPL/ONL junction as reference (C, *yellow*) appeared close and nearly parallel to each other, while the reference lines were further apart in areas of macular edema (left side of D, *magenta* and *yellow*). By using the OPL/ONL junction as the reference boundary, most of the anomalous INL flow signals were assigned to the ICP.

As there is a high frequency of macular edema in preterm infants and the macular edema is almost exclusively present in the INL,^22^^–^^25^ we first assessed if reference for the ICP/DCP boundary is better based on the IPL/INL or OPL/ONL junction. Following offset parameter optimization, we determined either 17.48 µm below the IPL/INL junction or 34.96 µm above the OPL/ONL junction as potential offsets for the ICP/DCP boundary. In infant eyes without macular edema, the reference junction did not significantly alter the vascular pattern of ICP and DCP (Fig. 1A, 1C). However, in the infant eye with macular edema, different vascular patterns of ICP and DCP were observed within the area of macular edema (Fig. 1B, 1D). The ICP slab was better visualized by using the IPL/INL junction as reference while the DCP slab was better visualized by using the OPL/ONL junction as reference. Given that the anomalous INL perifoveal microvasculature appeared more connected to the ICP than the DCP, we elected to assign it to the ICP. Therefore, we used the OPL/ONL junction as the reference layer in all three infants in this group.

**Figure 2.**
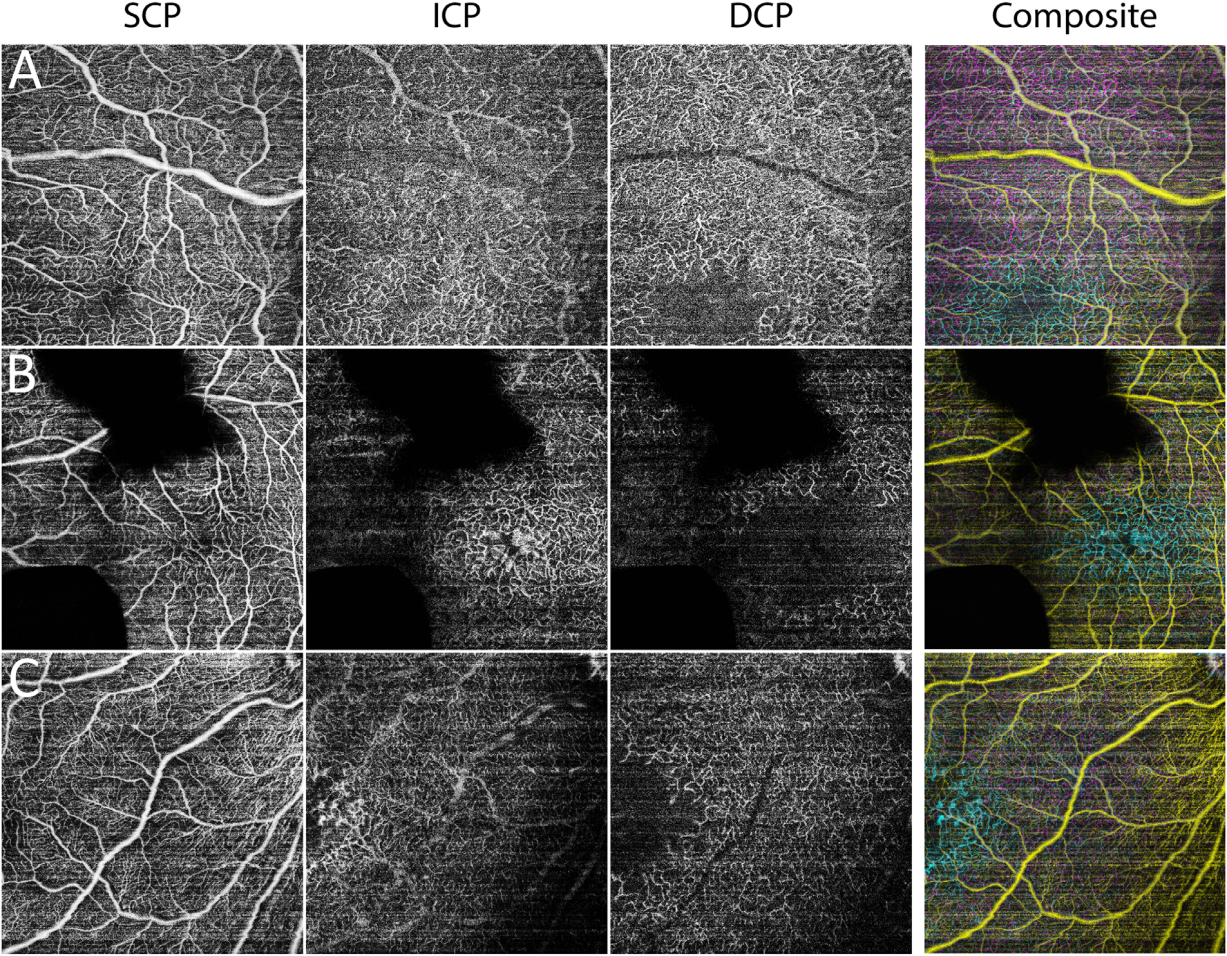
Segmentation of three-layered perifoveal retinal vasculature in three preterm infants imaged at around 40 weeks postmenstrual age. The larger arterioles and venules were almost exclusively located at the SCP; the parafoveal ICP exhibited a closed, loop-like vascular pattern; and the parafoveal DCP exhibited singular dendritic-like processes that extend toward the fovea. The area of signal loss in B was caused by blockage from vitreous hemorrhage. In the infant with macular edema, anomalous perifoveal endbulb-like flow signals could be seen in the ICP (C). A colored composite for the three capillary plexuses was generated and shown in the right-most column (SCP: *yellow*; ICP: *cyan*; and DCP: *magenta*).

**Figure 3.**
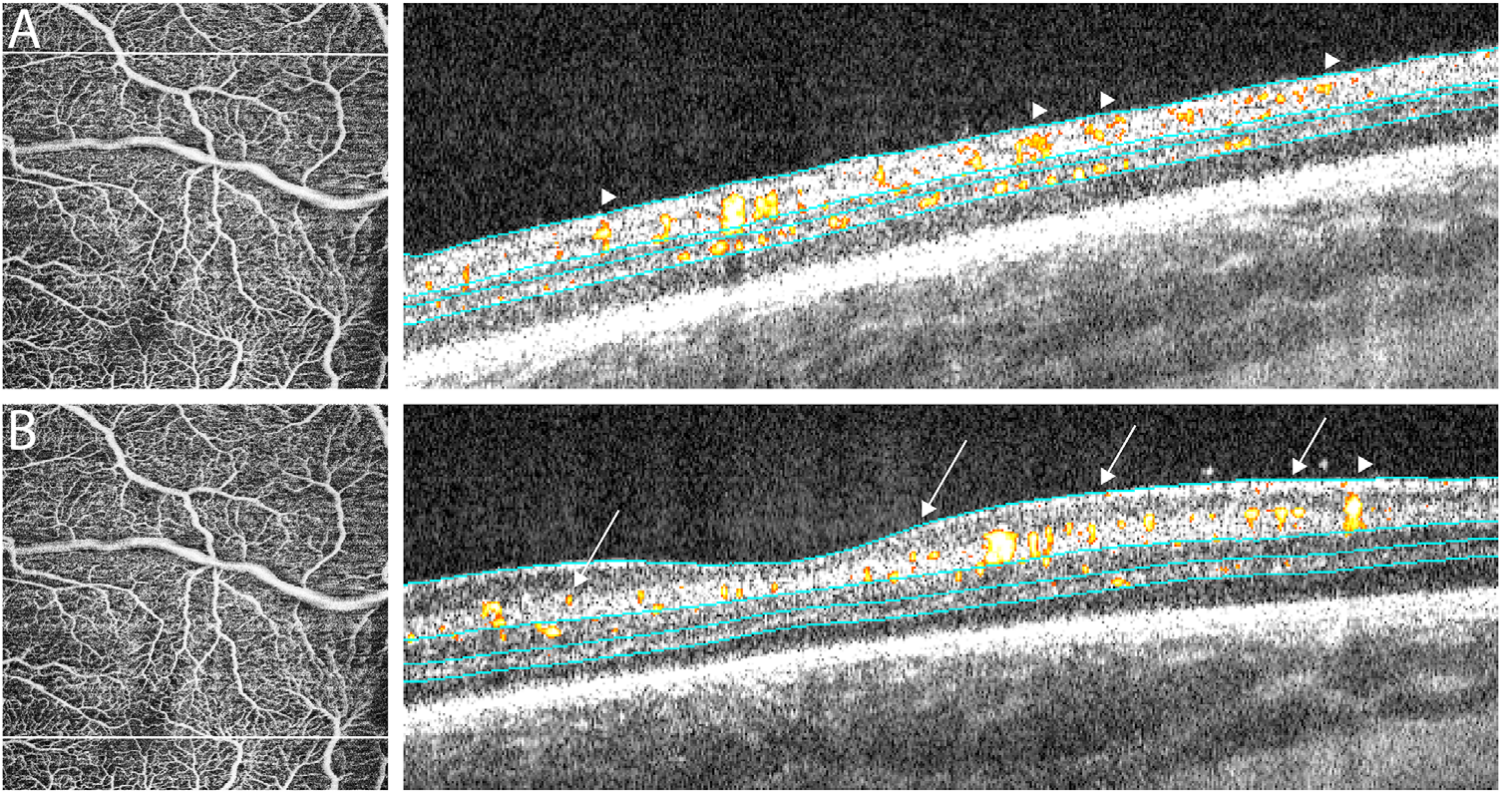
Cross-sectional B-scans with flow overlay in an infant eye at 40 weeks PMA showed that vertical location of the SCP varied based on its proximity to the fovea. Distal to the fovea, the SCP was present throughout the GCL (A and *right side* of B, *arrowheads*). In contrast, closer to fovea, the SCP was located closer to the GCL/IPL junction (B, *arrows*). Of note, the DCP terminated further from the fovea compared to the other capillary plexuses. *Cyan lines* represented the segmentation boundaries of the SCP, ICP, and DCP.

Applying our custom offset parameters, a three-layered retinal vascular structure was successfully visualized in all three eyes in the 40 weeks PMA group (Fig. 2). Each infant perifoveal capillary plexus was noted to have its characteristic vascular features: (1) large arterioles and venules were almost exclusively located in the SCP; (2) the ICP exhibited a closed, loop-like vascular pattern around the parafovea; and (3) the DCP displayed singular dendritic-like processes around the parafovea that extended toward the fovea. On cross-sectional B-scan with flow overlay, the SCP was largely present at the ganglion cell layer (GCL)/IPL junction proximal to the fovea (Fig. 3B) and throughout the GCL in areas more distal to fovea (Fig. 3A).

### Retinal Capillary Visualization Prior to Complete Secondary Vasculature Formation

**Figure 4.**
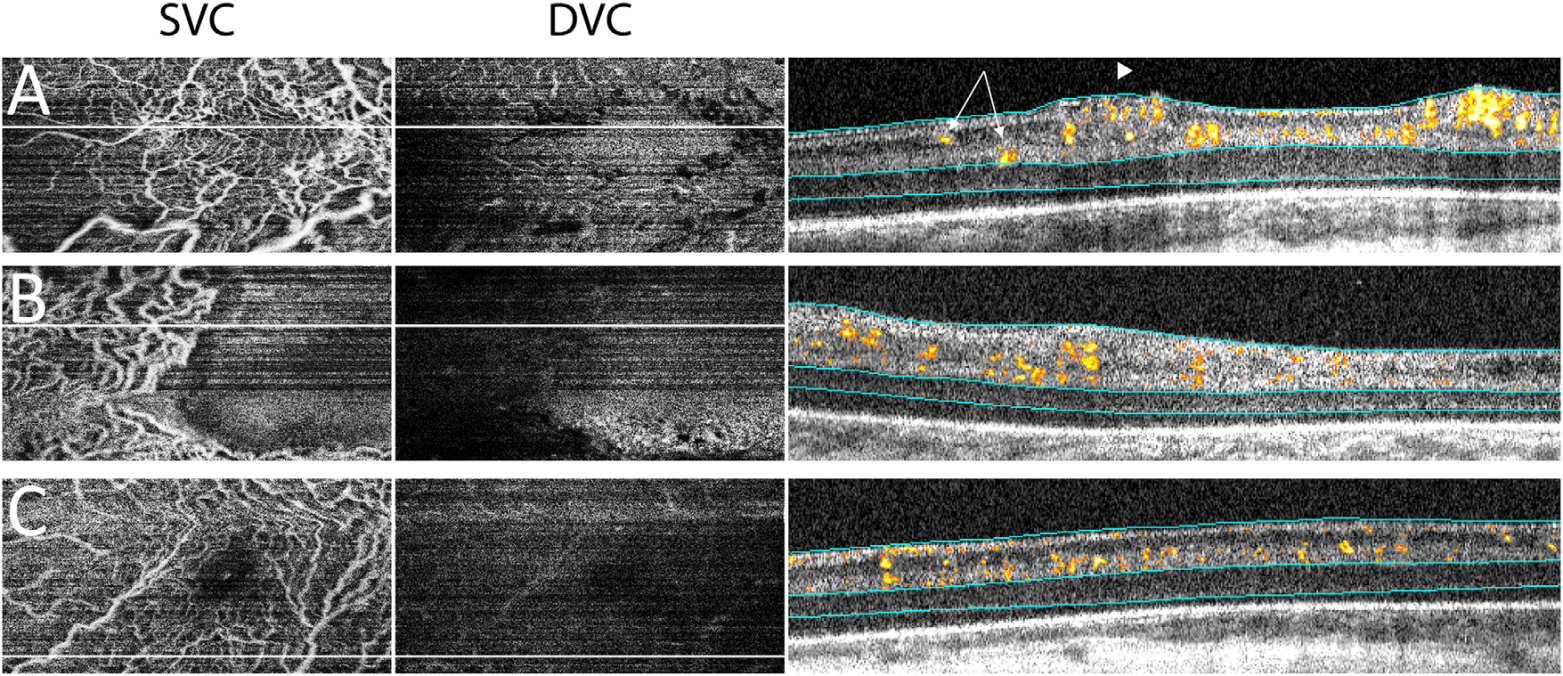
Segmentation of two-layered perifoveal retinal vasculature in three preterm infants imaged at around 35 weeks postmenstrual age. The SVC in these three infant eyes exhibited vascular dilation and tortuosity, while the DVC was largely avascular. Cross-sectional B-scans with flow overlay showed that the flow signals in all three eyes were largely above the IPL/INL junction. Scant flow signal along the IPL/INL junction may represent early deep vascular penetration (A, *arrows*). In one infant, neovascularization and inner retinal vessel protrusion were present, but the inner limiting membrane was not breached (A, *arrowhead*). *Cyan lines* represented the SVC and DVC boundaries.

In the 35 weeks PMA group, the three distinct layers of perifoveal capillaries were not well appreciated. All three eyes developed type 1 ROP and possessed an SCP that displayed vascular dilation and tortuosity (Fig. 4). The ICP and DCP were not discernable (individual slab data not shown). Scant, early flow signals were observed in the inner aspect of the INL for one of the three infants in this group (Fig. 4A). This may represent early vascular penetration of the INL. Of note, the fovea was not evident on cross-sectional B-scans in two of three eyes (data not shown). Hence, in infant eyes without obvious secondary vasculature, we collectively segmented the ICP and DCP as one deep vascular complex (DVC) and assigned the superficial vascular complex (SVC)/DVC boundary at the IPL/INL junction (Fig. 4).

Therefore, we recommend the following offset parameters for perifoveal vascular plexus visualization in infant eyes:

1. At term-equivalent age and with formed secondary vasculature:
  a. SCP inner boundary: vitreous/ILM junction
  b. SCP/ICP boundary: 8.74 µm above the IPL/INL junction
  c. ICP/DCP boundary: 34.96 µm above the OPL/ONL junction
  d. DCP outer boundary: at the OPL/ONL junction
2. At younger age without obvious secondary vasculature formation:
  a. SVC inner boundary: vitreous/ILM junction
  b. SVC/DVC boundary: at the IPL/INL junction
  c. DVC outer boundary: at the OPL/ONL junction

## Discussion

In this study, we visualized depth-resolved perifoveal retinal vasculature in preterm infants using handheld OCT-A and optimized the segmentation parameters for two stages of retinal vascular development. We demonstrated the lack of flow in the deep perifoveal vasculature in the 35 weeks PMA group and the presence of more mature three-layered capillary structures at term age. These findings represent a first step toward using noninvasive imaging to elucidate perifoveal vascular development and pathology in preterm infants.

Our findings of the primary retinal vasculature, the SCP, in the preterm infants paralleled those observed in previous histologic studies in postmortem infants and nonhuman primates.^3^^,^^4^^,^^9^ In the 35 weeks PMA group of preterm infants, we observed well-defined SCP morphology in all three eyes before and after closure of the temporal notch. In contrast, we observed occasional flow signal at the inner INL in only one of three eyes. This may represent initial penetrating vascular branches to establish the deeper vascular layers. Of note, retinal vascular development was delayed in all three eyes in this group, as all had subsequently developed type 1 ROP, and early neovascularization could be observed in one eye. In the 40 weeks PMA group, the vertical location of the SCP vasculature was also similar to the histologic observations from primates.^3^^,^^4^ Along the horizontal meridian, the SCP vasculature predominantly existed at the GCL/IPL junction around the fovea; while distal to the fovea, the SCP vasculature was more commonly located throughout the GCL.

Our findings of the secondary retinal vasculature, the ICP and DCP, also paralleled what was observed in nonhuman primates. First, we observed two stages of perifoveal vascular development: a three-layered perifoveal retinal vasculature in the 40 weeks PMA group and an essentially absent secondary perifoveal vasculature in the earlier age group. Second, similar to what was observed in fetal primates and adult human retina, the avascular zone in DCP was larger than SCP and ICP in all three infants in the 40 weeks PMA group.^4^^,^^7^ Prior histology studies in primates suggested that the DCP is established before the ICP around the fovea.^2^^,^^4^ Additional depth-resolved longitudinal OCT-A images from preterm infants will allow us to further clarify if such a process is also observed in preterm infants.

To our knowledge, our study is the first attempt to segment the perifoveal retinal vasculature as three layers in preterm infants (PubMed searches using “OCT angiography” and “infant” and “segmentation” on March 23, 2021). The three-layered perifoveal retinal vasculature is well established in adult eyes.^6^^,^^7^ Campbell et al.^6^ segmented the ICP and DCP at the midpoint of the INL, while Park et al.^7^ generated thin slabs (0 µm and 30 µm below the IPL/INL junction for ICP and 45 µm and 60 µm below the IPL/INL for DCP) using IPL/INL as the reference boundary. Neither method was perfect for the infant eyes. The high frequency of macular edema, as evident by cystoid spaces predominantly located in the INL, presents a unique challenge in determining the segmentation between ICP and DCP in preterm infants.^22^^–^^25^ We assessed both the IPL/INL junction and OPL/ONL junction as reference boundaries for segmentation of the ICP and DCP. When macular edema was not present, the vascular pattern of ICP and DCP was similar, irrespective of IPL or OPL as the reference boundary of choice. However, when macular edema was present, using IPL or OPL as the reference boundary generated different ICP and DCP vascular patterns in the area of cystoid spaces. This is largely due to the aberrant vascular structures located in the middle of the INL. Thus, depending on which layer the majority of the INL was ascribed to, these anomalous structures were designated to either ICP or DCP. We chose OPL/ONL as the reference boundary for segmenting the ICP and DCP because the DCP naturally possesses a larger foveal avascular zone and the anomalous vasculature around the fovea appeared more connected to the ICP. Future investigations and longitudinal studies will further clarify the designation of the ICP/DCP boundary.

Retinal vascular development in preterm infants may not recapitulate its physiologic process in utero. Indeed, five of the six preterm infants included in our study were born at a gestational age of ≤26 weeks. It is well known that the foveal avascular zone of children and adolescents who were born prematurely is small or absent.^26^^,^^27^ Retinal vascular development in preterm infants may be further complicated by the presence of ROP. As all infants in our 35 weeks PMA group developed type 1 ROP, it is likely that the lack of ICP and DCP in these patients is a manifestation of extreme prematurity, ROP, or both.

This study is limited by a small sample size and its cross-sectional nature. Despite improvement in image acquisition speed, handheld OCT-A remained technically challenging for imaging preterm infants and is often affected by both hand motion and infant eye motion. Our proposed offset parameters for segmenting infant OCT-A images may also be specific to our current patient population and study device. However, our findings represent an initial guideline as we venture into the depth-resolved visualization of the developing infant retinal vasculature.

## Conclusion

Our exploratory pilot study showed that it is possible to image and visualize depth-resolved perifoveal retinal vasculature in preterm infants. This represents a first step toward an imaging-based understanding of retinal vascular development and depth-resolved vascular pathologies of ROP.

## Supplementary Material

Supplement 1
