# Supplementary material for: Depth-Resolved Visualization of Perifoveal Retinal Vasculature in Preterm Infants Using Handheld Optical Coherence Tomography Angiography
Source: Transl Vis Sci Technol. 2021 Aug 6;10(9):10. doi: 10.1167/tvst.10.9.10 (PMC8356566; doi:10.1167/tvst.10.9.10)
Supplement: Supplement 1 [file tvst-10-9-10_s001.pdf]

Supplementary Material
Patel et al.

Supplementary Figure S1. Photo of imaging an infant in the nursery using the investigational handheld swept source OCT angiography device.

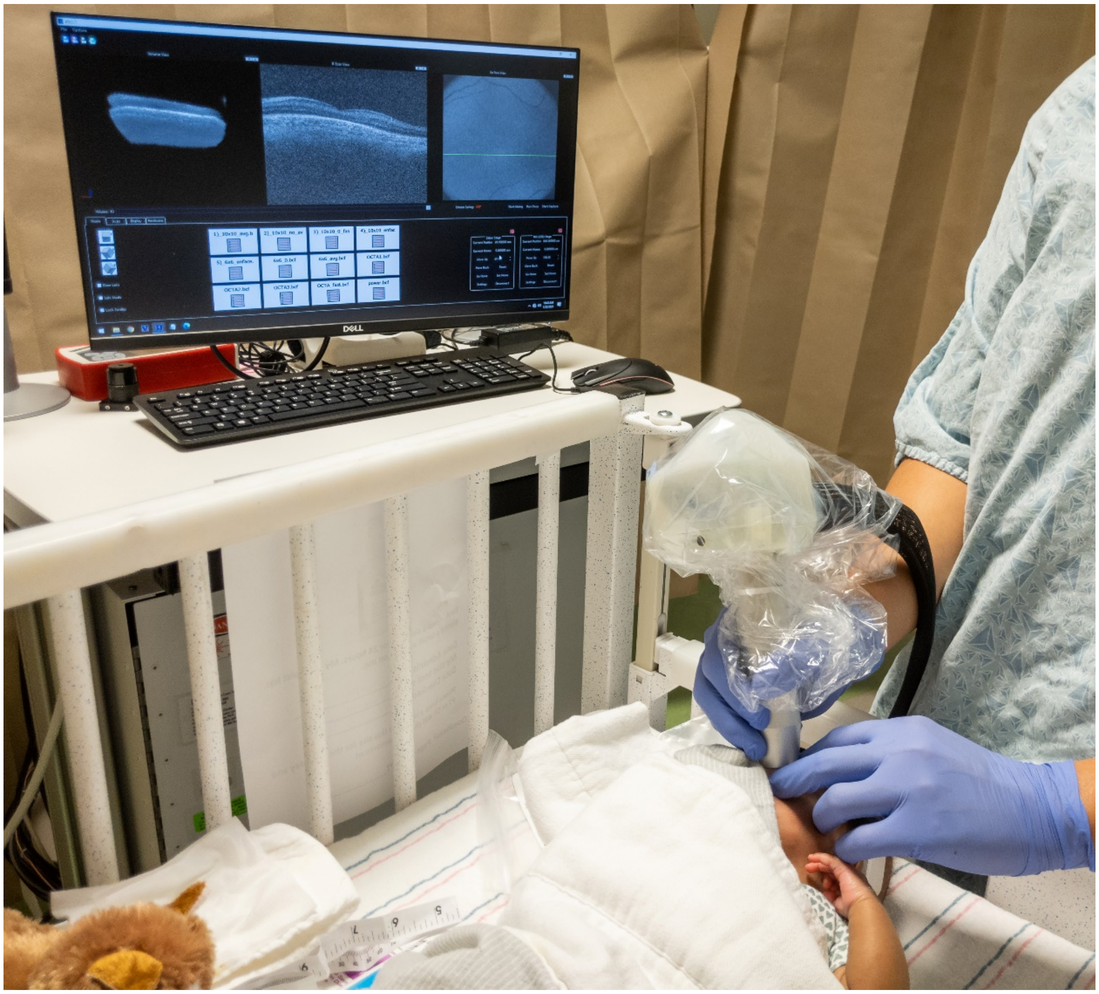

Supplementary Figure S2. A sample 25-slab collage of different offset parameters that were used to qualitatively assess the intermediate capillary plexus (ICP) in a preterm infant eye at 40 weeks postmenstrual age using the inner plexiform layer (IPL)/inner nuclear layer (INL) junction as the reference boundary. Each slab was marked by two numbers. The first number (preceded by a "+") within the bracket denoted the number of pixels above IPL/INL junction (i.e. superficial capillary plexus (SCP)/ICP boundary). The second number (preceded by a "-") within the bracket denoted the number of pixels below the IPL/INL junction (i.e. ICP/deep capillary plexus (DCP) boundary). The cyan and magenta rectangles represent the subsets of slabs (rows and columns, respectively) as presented to the graders. The yellow box denotes the optimal offset parameters that were selected by consensus. In addition to the IPL/INL junction, the ICP/DCP boundary was also assessed using the outer plexiform layer (OPL)/outer nuclear layer (ONL) junction as reference (data not shown).

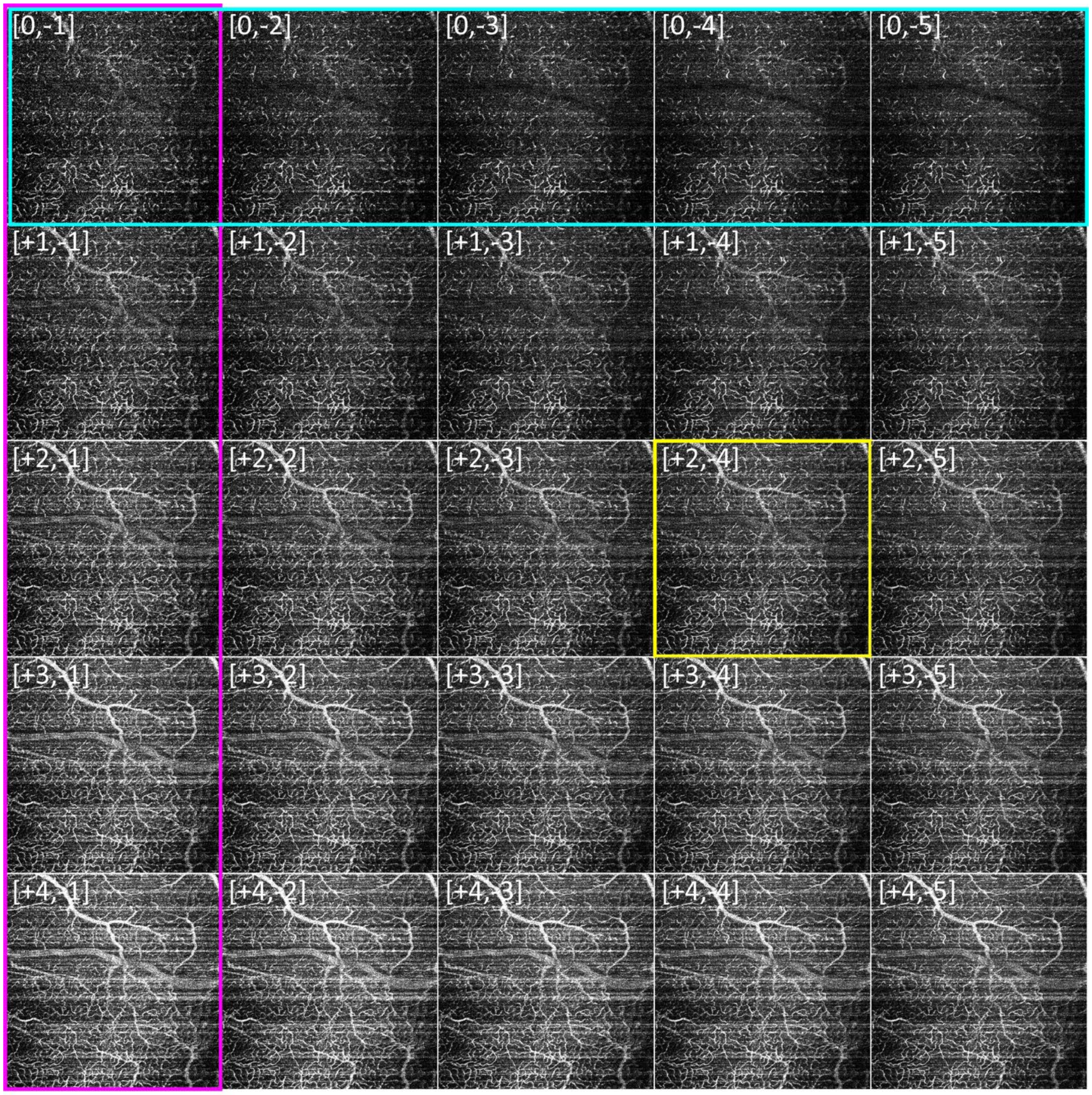

[0,-1]
[0,-2]
[0,-3]
[0,-4]
[0,-5]
[+1,-1]
[+1,-2]
[+1,-3]
[+1,-4]
[+1,-5]
[+2,-1]
[+2,-2]
[+2,-3]
[+2,-4]
[+2,-5]
[+3,-1]
[+3,-2]
[+3,-3]
[+3,-4]
[+3,-5]
[+4,-1]
[+4,-2]
[+4,-3]
[+4,-4]
[+4,-5]
